# Supplementary material for: Molecular design principles of Lysine-DOPA wet adhesion
Source: Nat Commun. 2020 Aug 4;11:3895. doi: 10.1038/s41467-020-17597-4 (PMC7403305; doi:10.1038/s41467-020-17597-4)
Supplement: Supplementary file 1 — Supplementary Information [file 41467_2020_17597_MOESM1_ESM.pdf]

Supporting Information for

Molecular Design Principles of Lysine-DOPA Wet Adhesion

Yiran Li[1,2§], Jing Cheng[1§], Peyman Delparastan[1§], Haoqi Wang[2§], Severin J. Sigg[1], Kelsey G. DeFrates[1], Yi Cao[2] and Phillip B. Messersmith[1,3*]

[§]These authors contributed equally.

[1] Departments of Bioengineering and Materials Science and Engineering, University of California, Berkeley, CA, USA

[2] Department of Physics, Nanjing University, Nanjing 210093, P. R. China.

[3] Materials Sciences Division, Lawrence Berkeley National Laboratory, Berkeley, CA, USA

*Contact Author:
philm@berkeley.edu
210 Hearst Memorial Mining Building
Department of Materials Science and Engineering
Berkeley, CA, USA 94720-1760
510-643-9631

Table of Content:

1. **Supplementary Methods**
2. **Supplementary Figure 1.** Cantilever modification
3. **Supplementary Figure 2.** Peptide synthesis
4. **Supplementary Figure 3**. MALDI spectra of the peptides
5. **Supplementary Figure 4.** Analytical HPLC curves of the peptides
6. **Supplementary Figure 5.** ^{1}H NMR spectra of KF (a) and KY (b) in D_2O
7. **Supplementary Figure 6.** ^{1}H NMR spectra of $(KF)_3$ (a) and $(KY)_3$ (b) in D_2O
8. **Supplementary Figure 7.** ^{1}H NMR spectra of $(KY)_{10}K$ in D_2O
9. **Supplementary Figure 8.** ^{1}H NMR spectra of $(KYP_8)_5KY$ in D_2O
10. **Supplementary Figure 9.** Contour length distribution
11. **Supplementary Figure 10.** Representative discarded F-X curves
12. **Supplementary Figure 11.** Representative F-X curves for interaction of $(KY)_3$ with TiO_2
13. **Supplementary Figure 12.** Representative F-X curves for interaction of $(KY)_{10}$ with TiO_2
14. **Supplementary Figure 13.** Representative F-X curves and rupture force distributions for interaction of $(KF)_3$ and $(KY)_3$ with PS and TiO_2 substrates
15. **Supplementary References**

Materials and methods:

Materials:

Fmoc-DOPA(acetonide)-OH was purchased from Chempep Inc. N_3-P_6-COOH and Fmoc-amido-P_8-acid were purchased from Broadpharm. Dibenzocyclooctyne-amine (DBCO), Fmoc-Lys(Boc)-OH, Fmoc-Phe-OH, Rink Amino MBHA resin, N,N,N′,N′-Tetramethyl-O-(1H-benzotriazol-1-yl)uronium hexafluorophosphate (HBTU), N,N-Diisopropylethylamine (DIPEA), 1,2-Ethanedithiol (EDT), Triisopropylsilane (TIPS), and a-cyano-4-hydroxy-cinnamic acid (CHCA) were purchased from Sigma-Aldrich. PBS powder concentrate was purchased from Fisher scientific. Sulfuric acid was from VWR and hydrogen peroxide was from Acros. Maleimide-PEG-NHS (5000 Da) was acquired from Nanocs Inc and maleimide-PEG-methoxy (2000 Da) was obtained from Jenkem.

Solid phase peptide synthesis:

Peptides were synthesized on an automated microwave peptide synthesizer (Liberty Blue, CEM) with Fmoc-strategy (**Scheme S1**). The Rink Amino MBHA resin (loading scale 0.2 mmol) was swelled in DMF for 30 min and ran through the following cycles of Fmoc-deprotection and amino acid coupling. **Fmoc-deprotection**: T = 75℃, power of microwave = 30 W, t = 5 min with piperidine (20 w% in DMF, 5 mL), three wash steps (DMF, 5 mL) after deprotection. **Amino acid coupling**: T = 75℃, power of microwave = 30 W, t = 10 min for Fmoc-Lys(Boc)-OH and 15 min for all other Fmoc-amino acid, with Fmoc-amino acid (0.2 M in DMF, 2 mL, 2 eq), HBTU (0.2 M in DMF, 2 mL, 2 eq) and DIPEA (1 M in DMF, 1 mL, 5 eq), two wash steps (DMF, 5 mL) after coupling.

N3-P6-COOH coupling:

Peptides on resin were deprotected in 20% of piperidine/DMF at RT for 30 min and then washed with 5 mL of DMF for three times. N3-P6-COOH (0.2 M in DMF, 1.1 mL, 1.1 eq), HBTU (0.2 M in DMF, 1.1 mL, 1.1 eq) and DIPEA (0.2 M in DMF, 4 mL, 4 eq) were added into the resins and shaken for 2 h (100 rpm, 37℃). The resins were washed by 5 mL of DMF for three times.

Resin cleavage:

Resin cleavage was performed with a mixture of TFA/DCM/EDT/TIPS (50:45:2.5:2.5, 10 mL) for 2 h. Peptides with 6 amino acid units or more were concentrated by rotavapor and precipitated from cold diethyl ether (90 mL), washed twice with diethyl ether and dried by N_2 blowing. Peptides with 2 amino acid units (KF and K<u>Y</u>) were concentrated by rotavapor and dried under high vacuum.

Purification and characterization:

Crude peptides were dissolved in 0.1% aqueous TFA to 10 mg/mL, and injected (2 mL/run) into a semi-preparative HPLC system. Analytical HPLC curves were obtained on Agilent 1260 Infinity Quarternary LC System with Vydac 218TP C18, 10 µm, ID 4.6 x 250 mm column, 0-100% B in 30 min gradient (A: water + 0.1% TFA, B: acetonitrile + 0.1% TFA), 1.00 mL/min flow rate, and 230 nm UV detector. After purification, the yields for peptides with 2 and 6 amino acids (KY, $(K\underline{Y})_3$, KF, $(KF)_3$) were ~10% (based on the ~0.7 mmol resin loading) after purification. The synthetic yields for longer chain peptides were roughly 2-3%.

Collected fractions were verified by MALDI spectra (Voyager DE Pro instrument) on reflector mode with CHCA as matrix, concentrated by rotavapor and lyophilized to obtain purified peptides. HPLC: Agilent 1260 Infinity Quarternary LC System, Column: ZORBAX SB-C18, 5 µm, ID 9.4 x 250 mm, Gradient: 0-15% B in 5 min, then 15-30% B in 20 min (A: water + 0.1% TFA, B: acetonitrile + 0.1% TFA), Flow rate: 4.00 mL/min.

Cantilever modification:

MLCT Silicon nitride cantilever (Bruker Nano Inc.) were first treated with piranha solution (H_2O_2:H_2SO_4=1:5 (v:v), Piranha is a very aggressive solution and should be used with caution) for 30 minutes. After rinsing with excessive DI water and gently drying under a stream of nitrogen, the cantilevers were transferred into 0.5% (v/v) MPTMS/toluene solution for 2 hours for thiol functionalization. The cantilevers were then rinsed with excess toluene to remove the unreacted MPTMS and placed in oven at 120˚C for 15 minutes to cure the alkoxysilane layer. Next the cantilevers were immersed in a 1:10 mixture of maleimide-PEG-NHS (5000 Da) and maleimide-PEG-methoxy (2000 Da), at a total concentration of 1 $mg.ml^{-1}$, in DMSO for 3 hours. This ratio was used to control the

binding density of bifunctional PEG and to reduce nonspecific interactions in the force spectroscopy measurements. The cantilevers were then rinsed with DMSO and incubated in a 0.5 mg.ml^{-1} solution of DBCO-amine in DMSO with 0.2% (v/v) trimethylamine for 2 hours. The cantilevers were washed with DMSO to remove unreacted reagent and were incubated in PBS (10 mM phosphate, 137 mM NaCl, pH 9) for 4 hours to hydrolyze maleimide-thiol bond to a stable ring-opened form. Finally, the cantilevers were immersed into 1 mg.ml^{-1} solution of peptides in DMSO for 1 hour. The modified cantilevers were then washed with DMSO and ethanol then dried under a stream of nitrogen.

Substrate preparation:

TiO_2 substrates were treated with Piranha solution for 30 minutes to remove organic residues from the surface. The substrates were then rinsed extensively with water and dried with nitrogen. Polystyrene substrates were cleaned by sonication in ethanol for 1 hour and then rinsed with excessive water and dried with nitrogen.

AFM-based force spectroscopy measurements:

AFM force spectroscopy measurements were carried out using a JPK ForceRobot 300 AFM (JPK Instruments AG, Germany). The experiments were performed in 10 mM PBS buffer (containing 137 mM NaCl) which was previously bubbled with nitrogen to degas dissolved oxygen to minimize catechol oxidation during the course of measurements. However, since the AFM chamber was exposed to air during force curve collection, it is expected that some oxygen was present in the buffer. Soft silicon nitride MLCT cantilevers of typical spring constant of 50-60 pN nm^{-1} were used for all experiments and calibrated using the thermal tune method after allowing the cantilever to equilibrate in solution for at least 30 minutes.[1] In a typical force measurement, the cantilever was approached to substrate at a constant speed of 1000 nm s^{-1} and held at the surface for 2 seconds to allow for the interaction between peptides and substrates. The cantilever was then retracted at the same speed. The force-extension curves were recorded using JPK data processing software and were further analyzed by a custom-written procedure in Igor Pro 6.12 (Wavemetric, Inc).

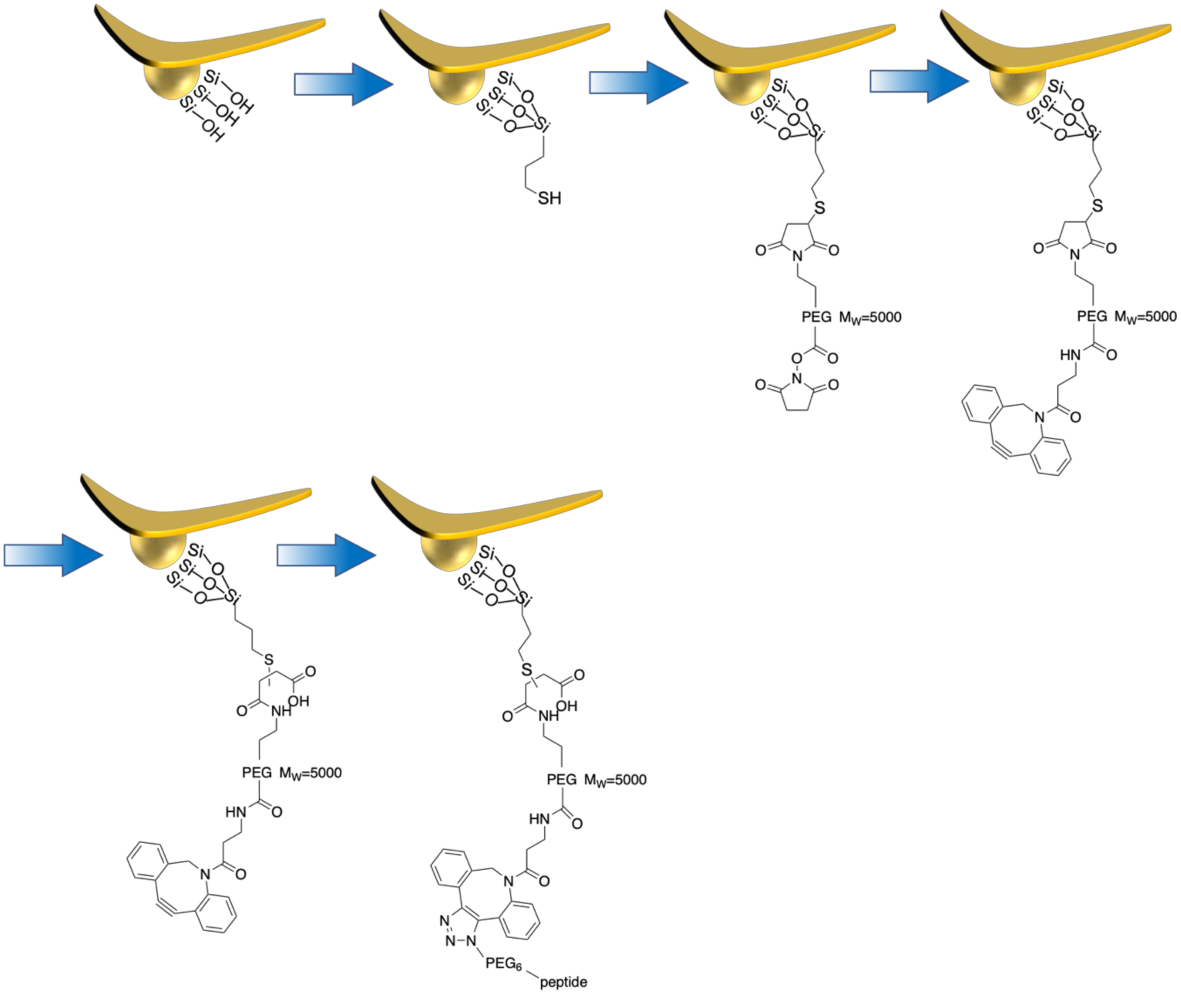

Supplementary Figure 1. Schematic of AFM cantilever modification steps (monofunctional methoxy-PEG is not shown).

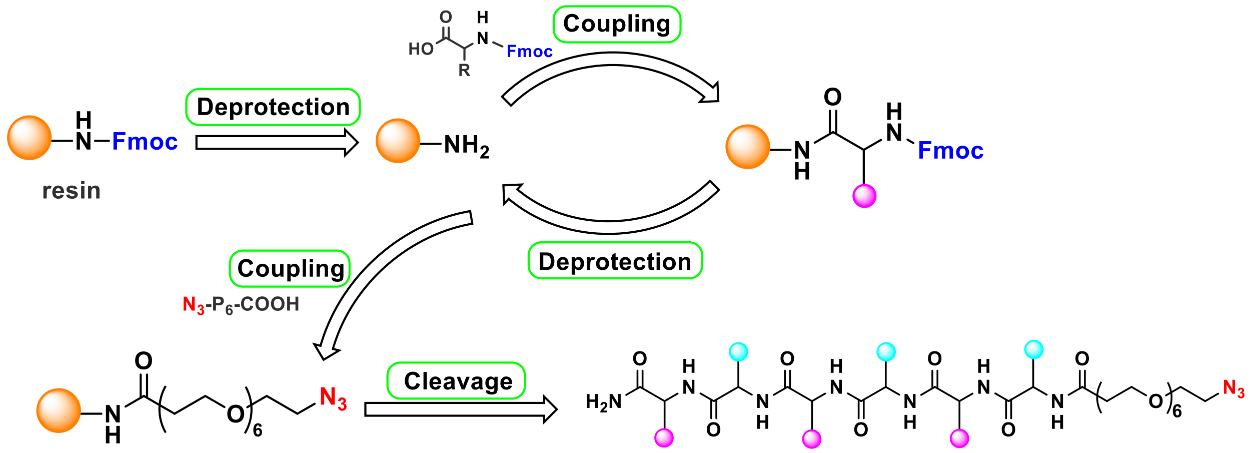

Supplementary Figure 2. Schematic of solid-phase peptide synthesis.

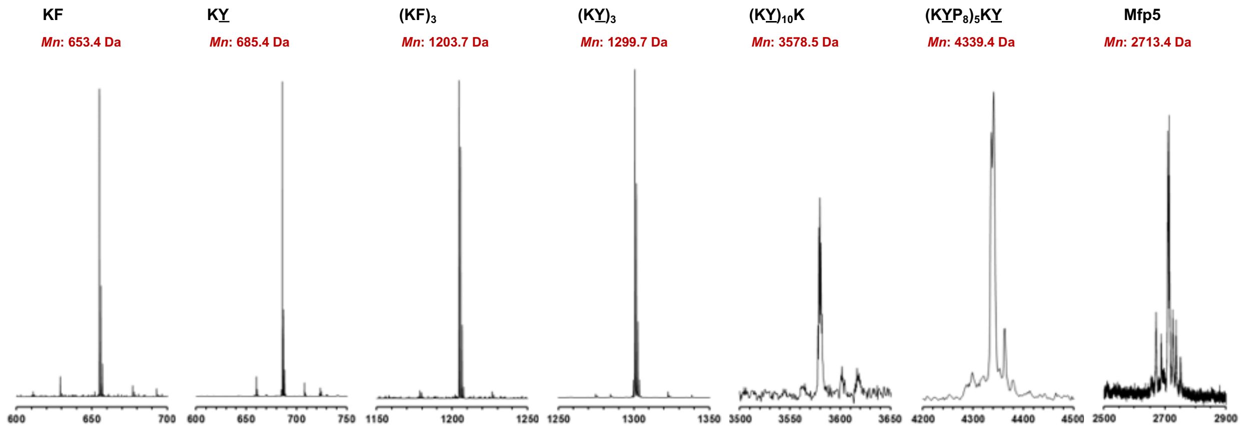

Supplementary Figure 3. MALDI spectra of the synthesized peptides. Measured mass is shown in red.

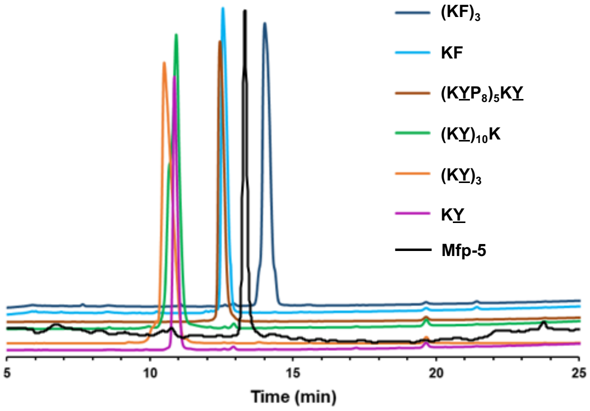

HPLC: C18 column, 1 mL/min, liner gradient ACN 0-100% for 30 min

Supplementary Figure 4. Analytical HPLC curves for the synthesized peptides.

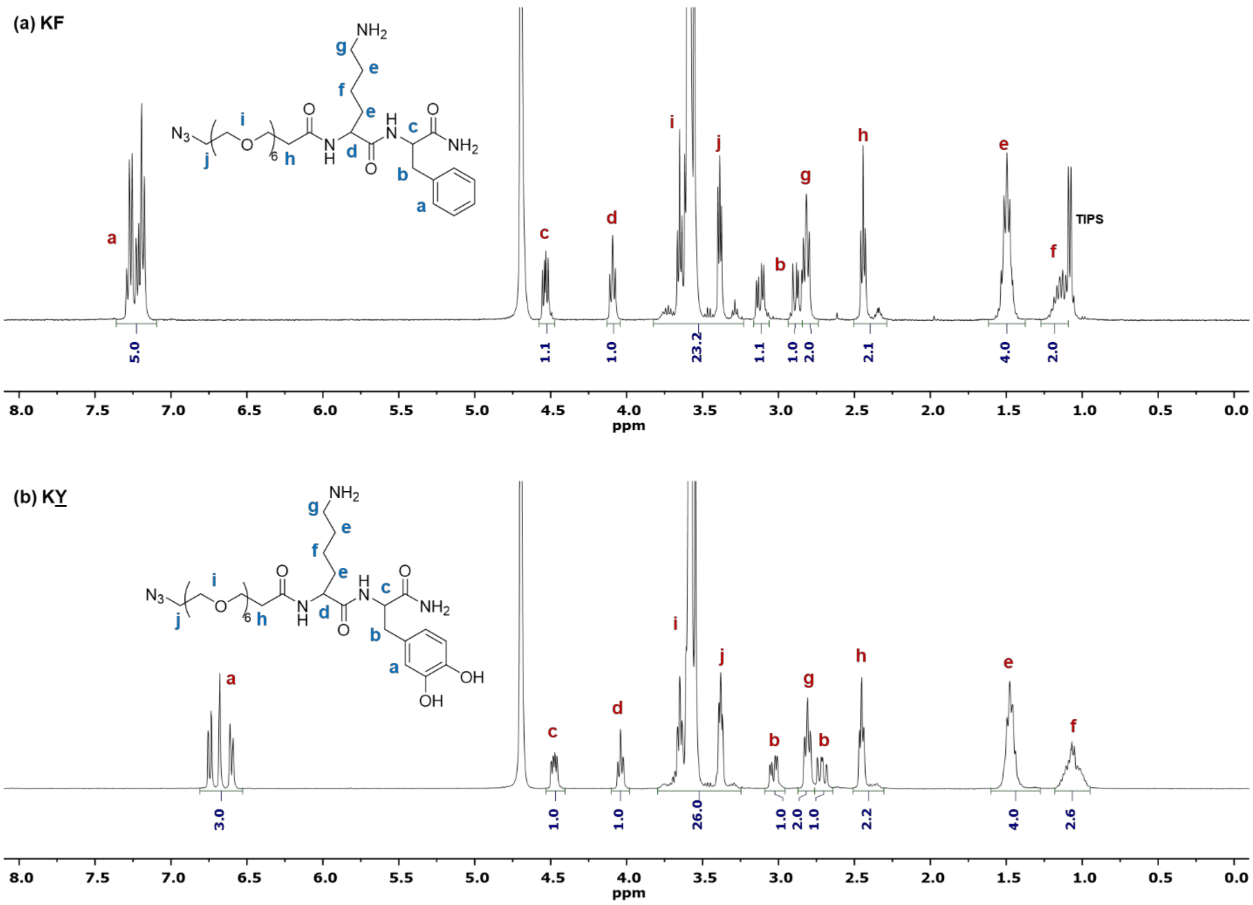

Supplementary Figure 5. ^{1}H NMR spectra of the peptides KF (a) and K<u>Y</u> (b) in D_2O.

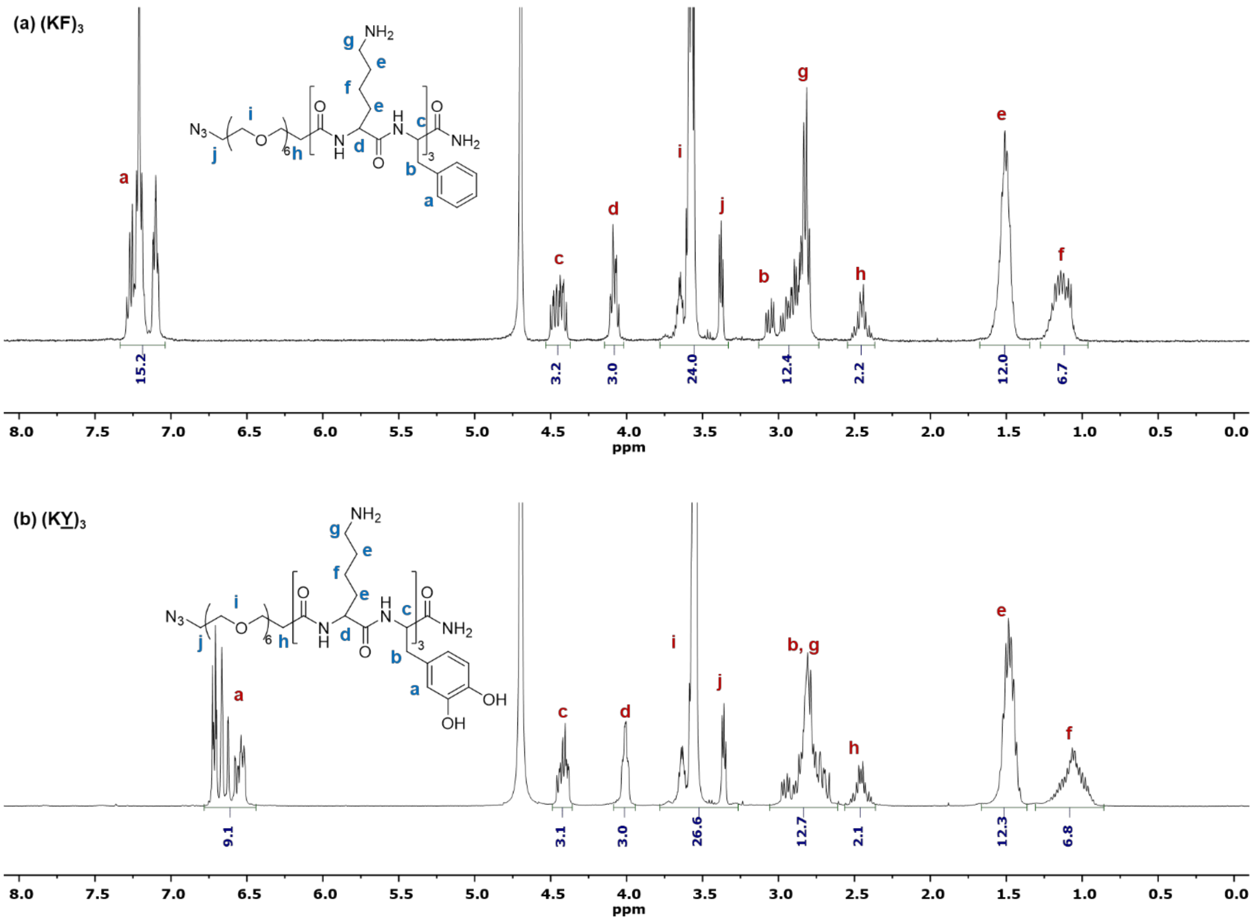

Supplementary Figure 6. ^{1}H NMR spectra of the peptides (KF)$_3$ (a) and (K<u>Y</u>)$_3$ (b) in D_2O.

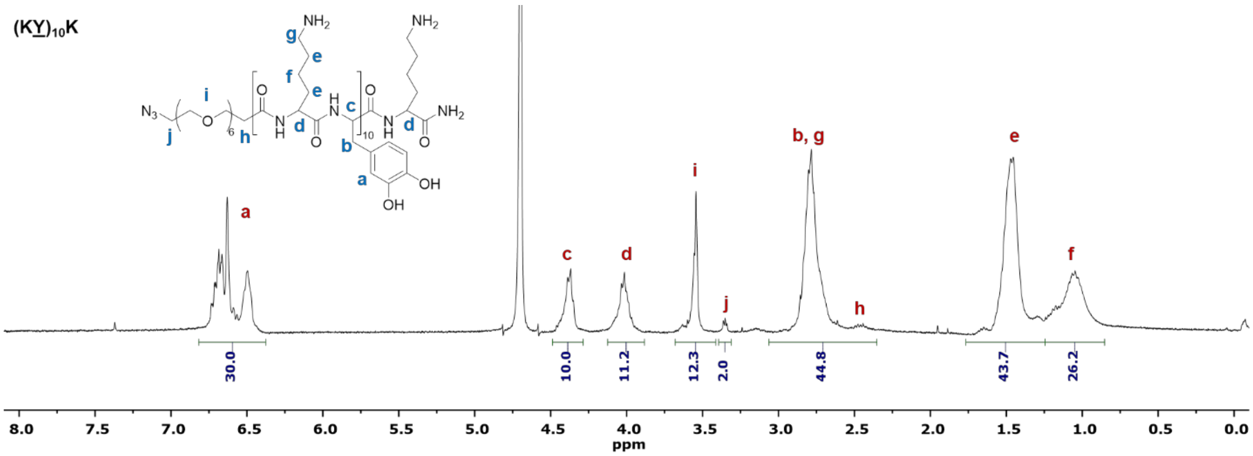

Supplementary Figure 7. ^{1}H NMR spectra of the peptide (K<u>Y</u>)$_{10}$K in D_2O.

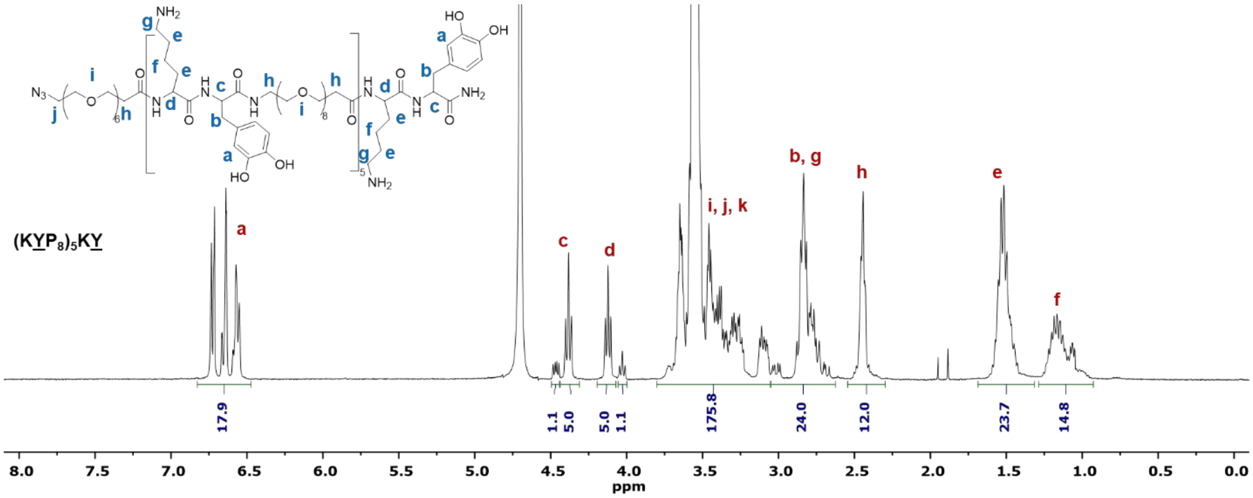

Supplementary Figure 8. ^{1}H NMR spectra of the peptide (K<u>Y</u>P8)5K<u>Y</u> in D_2O.

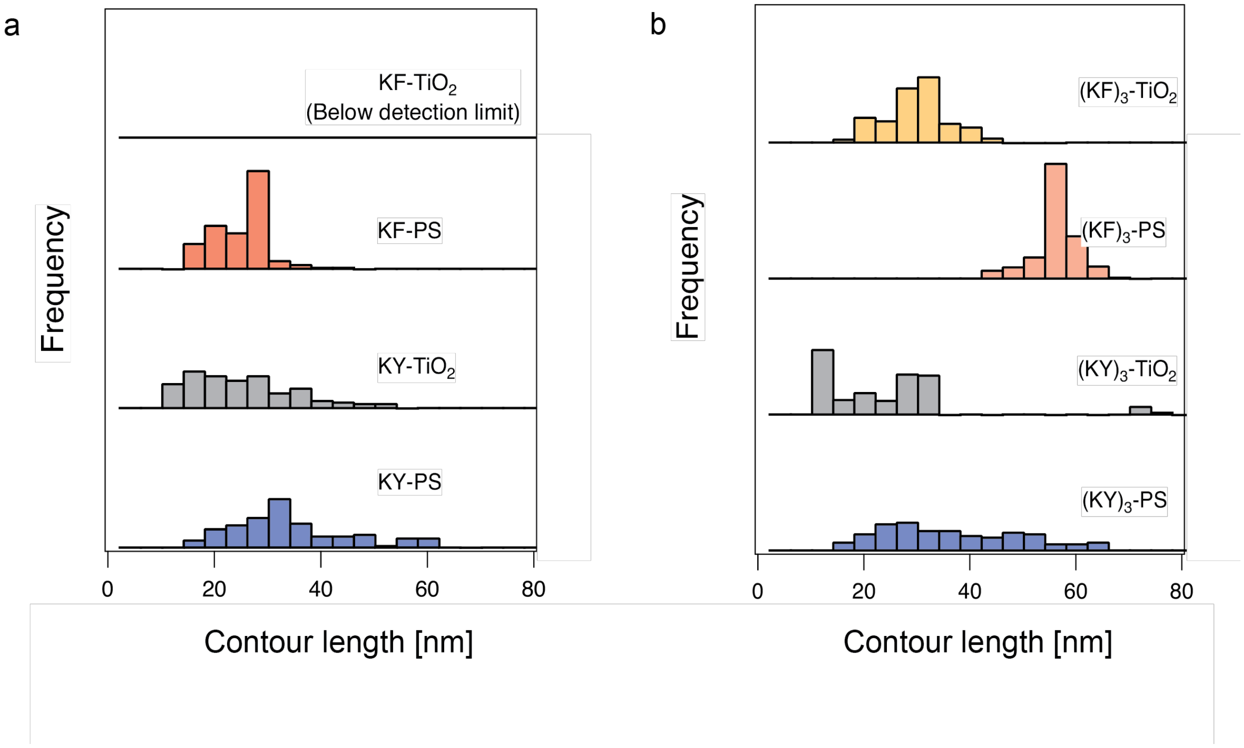

Supplementary Figure 9. Contour length distribution for rupture events; **(a)** for interaction of (K<u>Y</u>) or (KF) and **(b)** for interaction of $(K\underline{Y})_3$ or $(KF)_3$ with different surfaces. The distribution of contour lengths is attributed to the polydispersity of the PEG linker as well as randomness in the conjugation site on the cantilever tip. Source data are provided as a Source Data file.

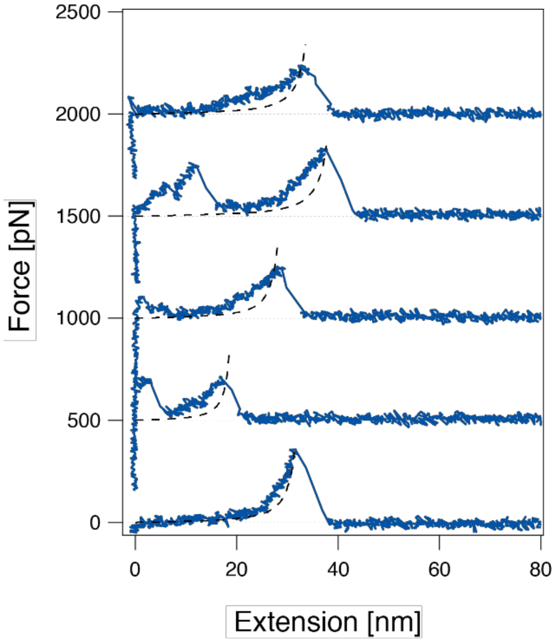

Supplementary Figure 10. Representative discarded F-X curves for the interaction of (KY) peptide with the TiO_2 surface. The black dashed lines correspond to the worm-like chain model fitting with persistence length of 0.36 nm. The force-extension curves whose fitted persistence length are larger than 0.4 nm or smaller than 0.32 nm were discarded and not included in the data analysis for calculating rupture force distribution. Source data are provided as a Source Data file.

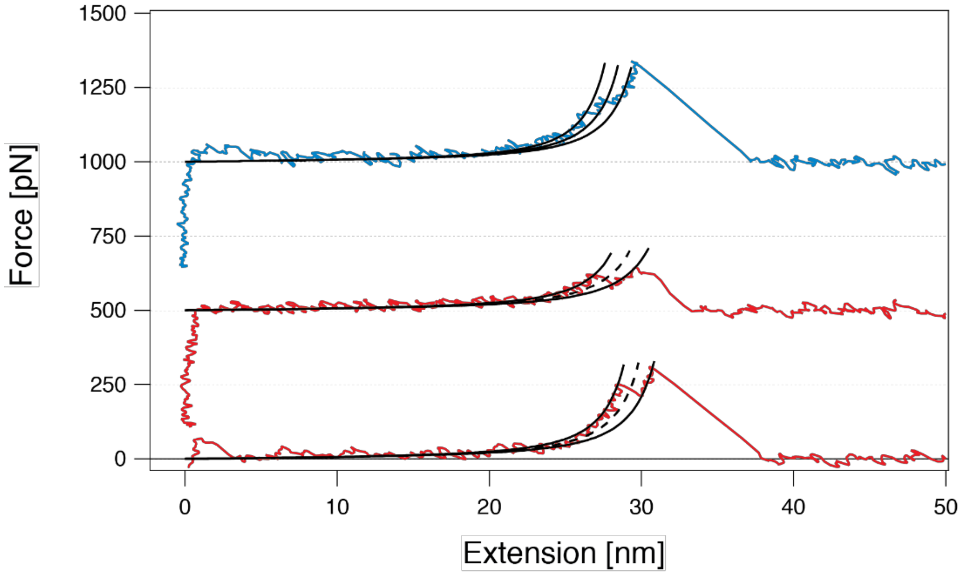

Supplementary Figure 11. Representative F-X curves for interaction of (KY)$_3$ peptide with TiO_2 surface. The black lines correspond to worm-like chain fitting with persistence length of 0.36 nm and the contour length increment of 1 nm. The blue curve shows three detachment events of (KY)$_3$ peptide from the surface. The dashed lines in the red F-X curves show a possible missing rupture event. As 1 nm contour length increment reaches the AFM detection limits, distinguishing three individual rupture peaks becomes difficult. Source data are provided as a Source Data file.

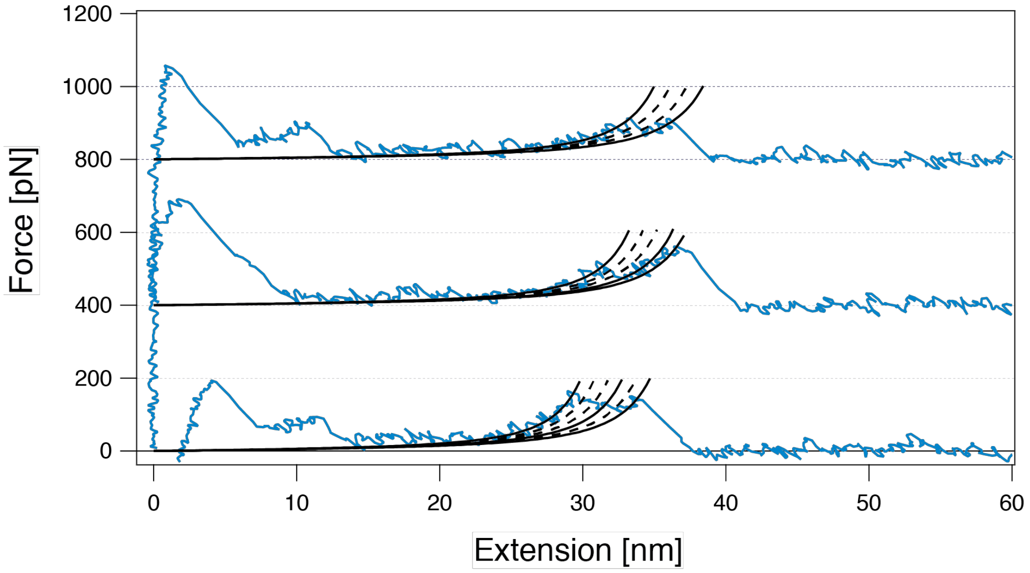

Supplementary Figure 12. Representative F-X curves for the (K<u>Y</u>)$_{10}$ peptide interacting with TiO_2 substrate. Black lines correspond to the worm-like chain fitting. Dashed lines represent the expected unbinding events based on the distance between adhesive moieties. Data indicates that that not all the adhesive sites were able to successfully attach to the surface. Source data are provided as a Source Data file.

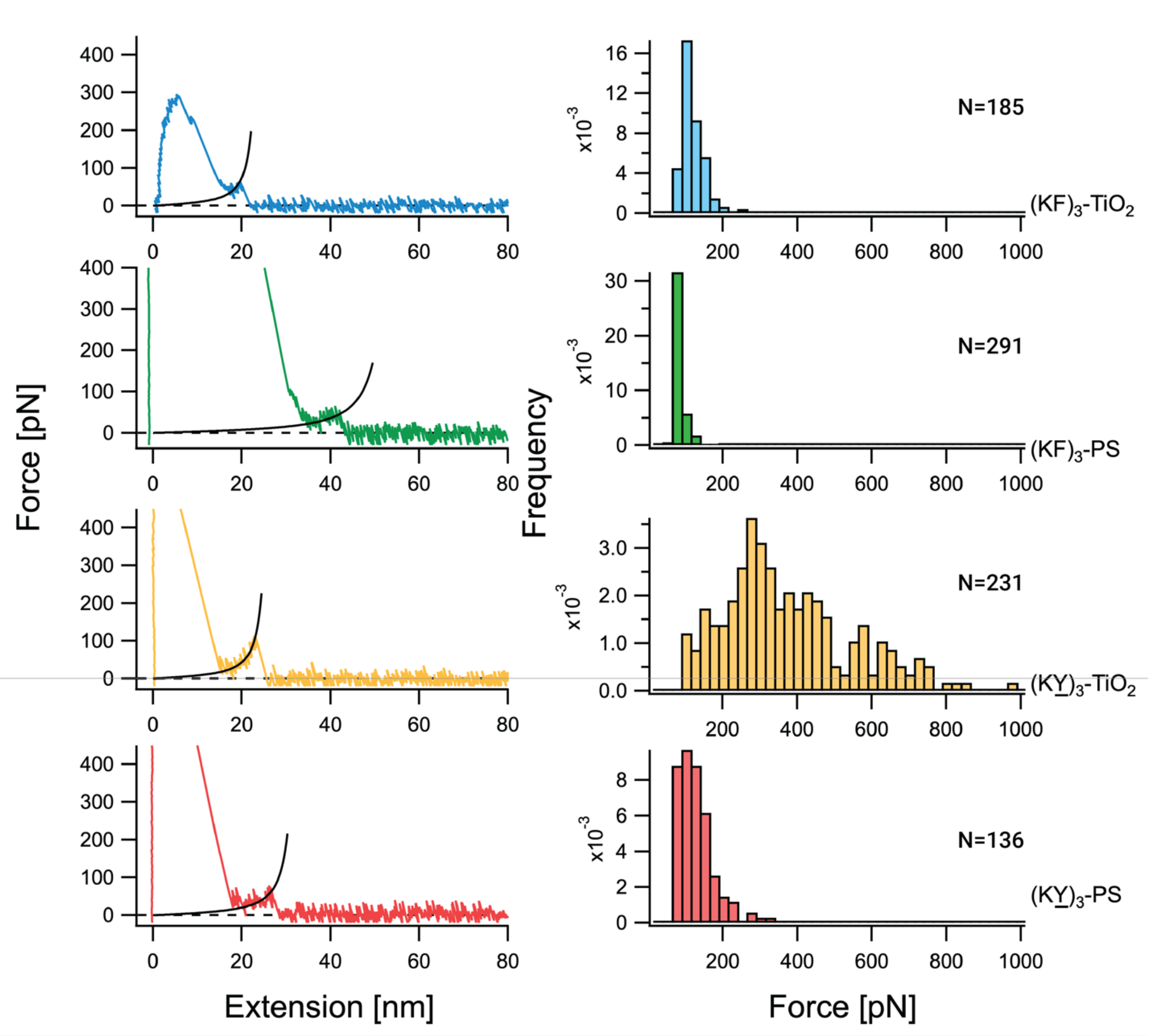

Supplementary Figure 13. Representative F-X curves (left) and rupture force distribution (right) for the interaction $(KF)_3$ and (K<u>Y</u>)$_3$ peptides with PS and TiO_2 substrates. N values represent the total number of rupture events used to plot the histograms. The black lines in the F-X curves correspond to the worm-like chain fitting. Source data are provided as a Source Data file.

Supplementary References:

1. Lubbe, J.; Temmen, M.; Rahe, P.; Kuhnle, A.; Reichling, M., Determining cantilever stiffness from thermal noise. *Beilstein J Nanotechnol* **2013,** *4*, 227-33.
2. Schlierf, M.; Li, H.; Fernandez, J. M., The unfolding kinetics of ubiquitin captured with single-molecule force-clamp techniques. *Proc Natl Acad Sci U S A* **2004,** *101* (19), 7299-304.
